# High-yield large scale production of size-customized microplastics from biodegradable plastic mulches via variable-speed mechanical milling

**DOI:** 10.1016/j.mex.2026.104110

**Published:** 2026-08-21

**Authors:** Mathilde Henrion, Ana M. Pelacho, Iseult Lynch, Lluis Martin-Closas

**Affiliations:** a Department of Agricultural and Forest Science and Engineering, University of Lleida, Av Alc. Rovira Roure, 191, Lleida, 25198, Spain; b School of Geography, Earth and Environmental Sciences, University of Birmingham, Birmingham, B15 2TT, UK

**Keywords:** Microplastics’ production, Biodegradable mulches, Milling, Size distribution, Micronisation

## Abstract

Biodegradable mulch films are increasingly used and incorporated into agricultural soils as an alternative to conventional polyethylene mulches. Although they are designed to reduce long-term plastic accumulation, they break down into microplastics before biodegradation, raising concerns about their potential ecotoxicological effects. Reliable risk assessment requires the evaluation of microplastics derived from the same mulch materials as used in the field. However, producing large quantities of microplastics from biodegradable mulch films across controlled size ranges remains challenging and often relies on costly cryogenic systems.

We present a rapid, high-yield, and cost-effective method for generating microplastics from commercial biodegradable mulch films using an ultra-centrifugal mechanical mill assisted by liquid nitrogen. Microplastics in the range from <0.2 to 5 mm were produced from 600 g of BDM, with an efficiency recovery over 96% of the milled mulch. This procedure prevents polymer softening and melting during fragmentation and enables reproducible micronisation with the milling speed controlling the size distribution directly and the proportion of smaller particles increasing with higher speeds. The method allows efficient production of hundreds of grams of microplastics within hours while minimising liquid nitrogen consumption.

• Cost-effective and high-yield production of microplastics from biodegradable mulch.
• Speed-dependent control of particle size distribution.
• An accessible alternative to dedicated cryogenic milling systems.

## Graphical abstract

**Figure:**
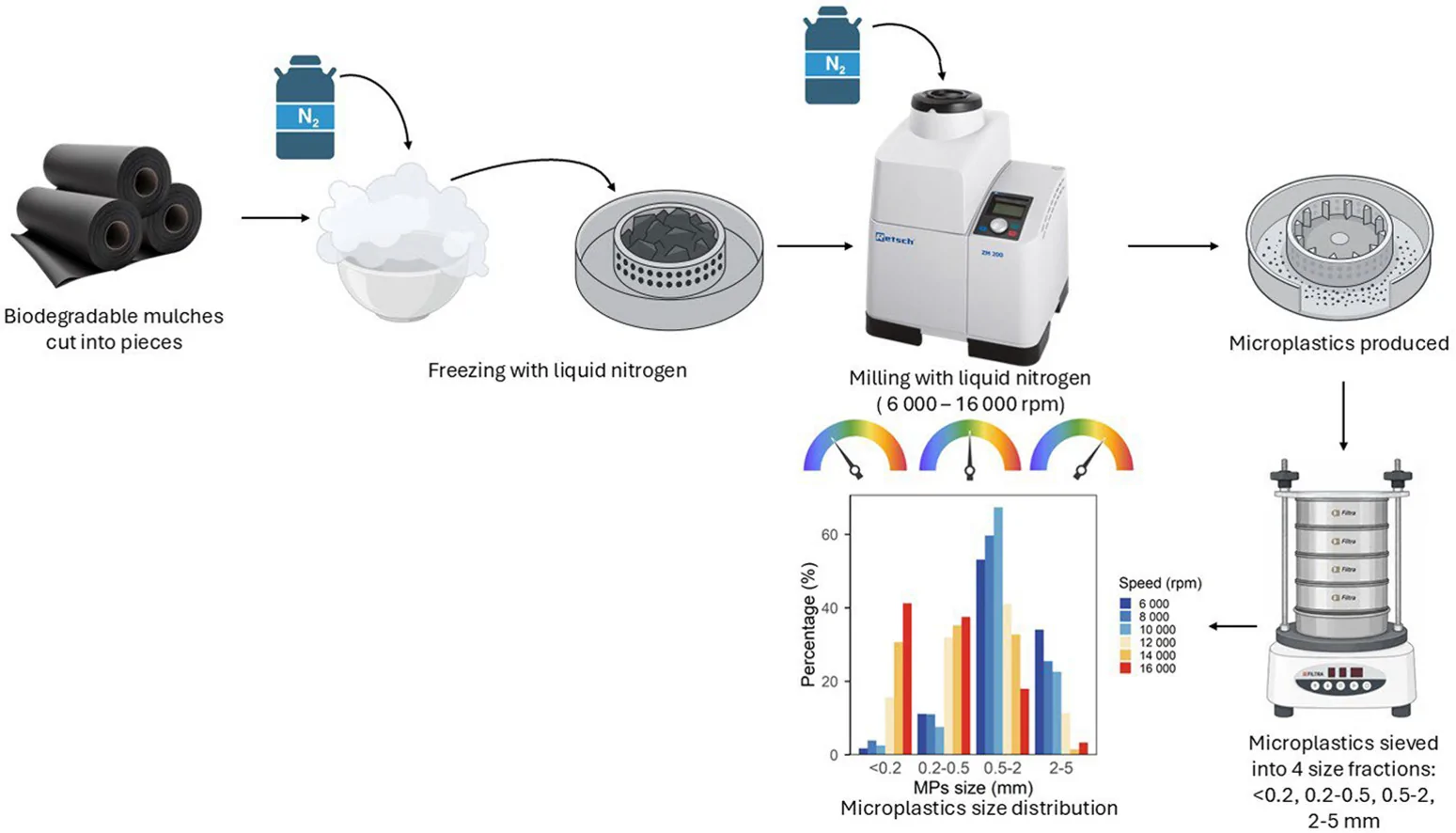

**Specifications table**

**Table:** 

**Subject area**	Environmental Science
**More specific subject area**	Environmental Pollution / Microplastics / Agricultural plastic mulches
**Name of your method**	Production of microplastics from biodegradable plastic mulches: High-yield and high-volume milling with speed-dependent size distribution
**Name and reference of original method**	NA
**Resource availability**	Liquid nitrogen Ultra-centrifugal mill (Retsch ZM200, Germany) https://www.fishersci.fr/shop/products/zm-200-model-ultra-centrifugal-mill/10786671 Vibratory sieving tower and sieves (FTS-0200, Filtra vibración, Spain) https://filtra.com/wp-content/descargas/IRIS-FTS-0200-English.pdf

## Background

Plastic mulching is a widely used agricultural technique that offers multiple benefits for improving crops, their management and sustainability [1,2]. Polyethylene (PE) plastic mulching, introduced in the late 1950s [3,4], has proven to be widely efficient. However, PE mulches do not degrade, and removing them is unfeasible resulting in the accumulation of microplastics (MPs, 1–5000 µm in size) in the soil [5]. Biodegradable mulches (BDM), defined as materials capable of breaking down and being metabolized by natural microorganisms and made of biodegradable aliphatic polyesters or starch-polymer blends [6,7], began to be developed in the late 1990s as a sustainable alternative to avoid plastic pollution, and have been commercially available since the early 2000s [3]. BDM are designed to be incorporated directly into the soil at the end of their useful life, where they weather and break down into smaller fragments over time, with the soil microflora converting them into carbon dioxide, water and biomass under aerobic conditions [8]. BDM can thus minimize the environmental impact of conventional plastic mulches which are highly persistent [6,[9], [10], [11]].

Although the biodegradability of the BDM represents a significant step forward over non-degradable plastic mulches, concerns have arisen due to their potential to generate a greater quantity of MPs than conventional PE films during the initial stages of their degradation [8]. MPs from BDM can alter soil carbon dynamics [12], with their effects varying depending on their abundance, size or residence time [13]. Hence, assessing the risks associated with BDM-derived MPs size and testing their effects on different organisms and matrices is urgently needed. However, the lack of availability of MPs from BDM formulations used in the field, including their additives, hampers the exploration of their impacts. Extracting and collecting BDM MPs from soils requires specific and time-consuming techniques [14] that are not feasible to collect the amounts needed for testing of their effects. A simple and efficient method to produce significant quantities of BDM MPs of various sizes is required in order to evaluate their size-specific effects in different experimental systems.

While cryogenic milling has been used to obtain small MPs [15,16], it is neither common nor affordable for many research laboratories. Mechanical mills are a widely available alternative. Their feasibility for producing MPs with liquid nitrogen has been demonstrated for coarse plastic litter collected on the beach, composed of conventional polymers [17]. However, the yield of MPs obtained was low, and poor for the larger particles in the size distribution [17]. BDM present additional challenges due to their biodegradable nature, low thickness (15 µm) and high elasticity, which significantly influence their fragmentation behaviour under milling, underscoring the need to develop a reliable micronisation methodology for BDM. Thus, we developed a low-cost, high-efficiency mechanical milling method assisted by liquid nitrogen to reproducibly produce size-fractionated BDM MPs. The size distribution of the MPs (up to 5000 µm) can be tailored by modulating the milling speed. The method has allowed us to demonstrate that the impacts of BDM MPs on plant development is size-dependent [18].

## Method details

### Materials and reagents

The BDM used in this study was a 15 µm-thick black commercial film, composed of poly(butylene adipate-co-terephthalate) (PBAT), corn starch and vegetable oils (Mater-Bi®, Novamont, Italy) with a density of 1.27 g/cm².

The final procedure requires cutting of the BDM into portions using metal scissors and cooling them in a mortar with liquid nitrogen.

Mixtures of MPs of varying particle sizes were produced using an ultra-centrifugal mill (Retsch ZM200, Germany) equipped with a 12-tooth rotor (radius of 4.95 cm) and a 4 mm sieve, operating at milling speeds ranging from 6000 to 16,000 rpm.

The size distribution of the MPs for each milling speed was determined using an electromagnetic sieve shaker (FTS-0200, Filtra Vibración, Spain) fitted with four metal sieves: 0.2, 0.5, 2.0 and 5.0 mm.

The produced MPs were stored in glass bottles, protected from light exposure.

### Preparatory

The final methodology for an efficient way to produce MPs of different size distributions from BDM consists of several steps where rapidity is crucial.

**Fig. 1:**
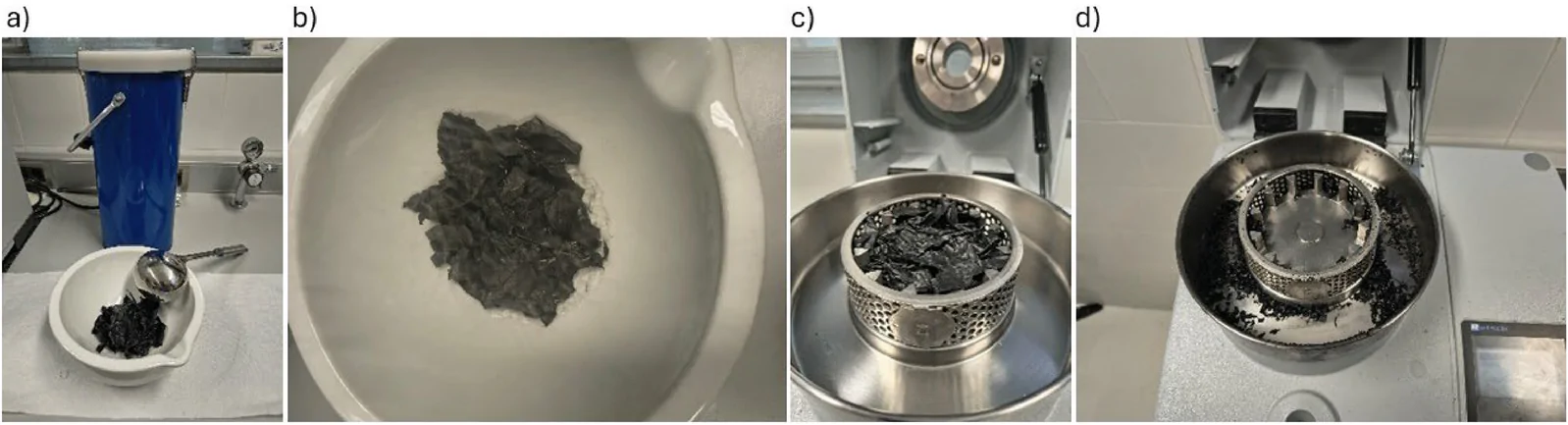
Steps applied to produce BDM-derived MPs with liquid nitrogen. a) freezing of the ∼20 cm^2^ BDM pieces, b) BDM pieces frozen using liquid nitrogen, c) pre-cooled mill sieve filled with the BDM fragments, d) BDM MPs produced after milling under liquid nitrogen for 7 s. Steps a-d are described in detail in the preparatory section.
(3) The pieces were transferred to the mill, which was previously cooled down with 50 mL of liquid nitrogen for 30 s (Fig. 1c). Keeping the mill at a low temperature is crucial to ensure efficient milling and to prevent the polymer from softening. It was noticed in previous preparatory tests that the equipment may warm due to friction from immediately previous milling runs. Under these circumstances, the polymer pieces tended to soften or partially melt, consistent with the transition of the polymer to a viscoelastic state as the temperature approaches or exceeds the glass transition temperature [7], producing fibrous fragments instead of MPs. Melting may also lead to sieve blockage, producing low yields and interruption of the milling process. Pre-cooling the mill with liquid nitrogen before each use effectively prevented heating and allowed consistent micronisation.
(4) The mill was immediately closed, an additional 50 mL ladle of liquid nitrogen was introduced from the top, and milling was started. The speed was selected from 6000 to 16,000 rpm. The milling was stopped just after the stabilisation of the speed, with the total milling time being around 7 s (Fig. 1d).

(1) The film was randomly cut into smaller pieces (maximum 20 cm²) with metal scissors. This size enables homogeneous freezing by liquid nitrogen in a 1 L mortar and facilitates transfer to the mill.
(2) The mortar was cooled down with 100 mL of liquid nitrogen for 30 s before adding 5 g of mulch pieces, followed by two sequential additions of 50 mL of liquid nitrogen at 10–15 s intervals (Fig. 1a). The material turned from black to grey within a few seconds and the pieces wrinkled (Fig. 1b). By these two qualitative criteria, it was identified that the material was sufficiently frozen to proceed with the process.

The mill is cleaned before and after each operation. All parts are first cleaned with hot distilled water and soap, rinsed twice with distilled water and allowed to dry. Subsequently, the components are rinsed with acetone to remove any remaining organic residues. After cleaning, the mill is used to produce MPs following the procedure described above. Upon completion of the operation, residual MPs are removed from the mill using a natural bristle brush followed by vacuum cleaning. The complete cleaning procedure is repeated before the next milling run.

The size sieving following the milling was effective in classifying the MPs into size fractions from lower than 0.2 mm; 0.2 to 0.5 mm; 0.5 to 2 mm and 2 to 5 mm, to assess the size distribution produced at each milling speed. Other sieve sizes may be freely selected to accommodate the targeted particle sizes required for the intended tests.

## Method validation

**Fig. 2:**
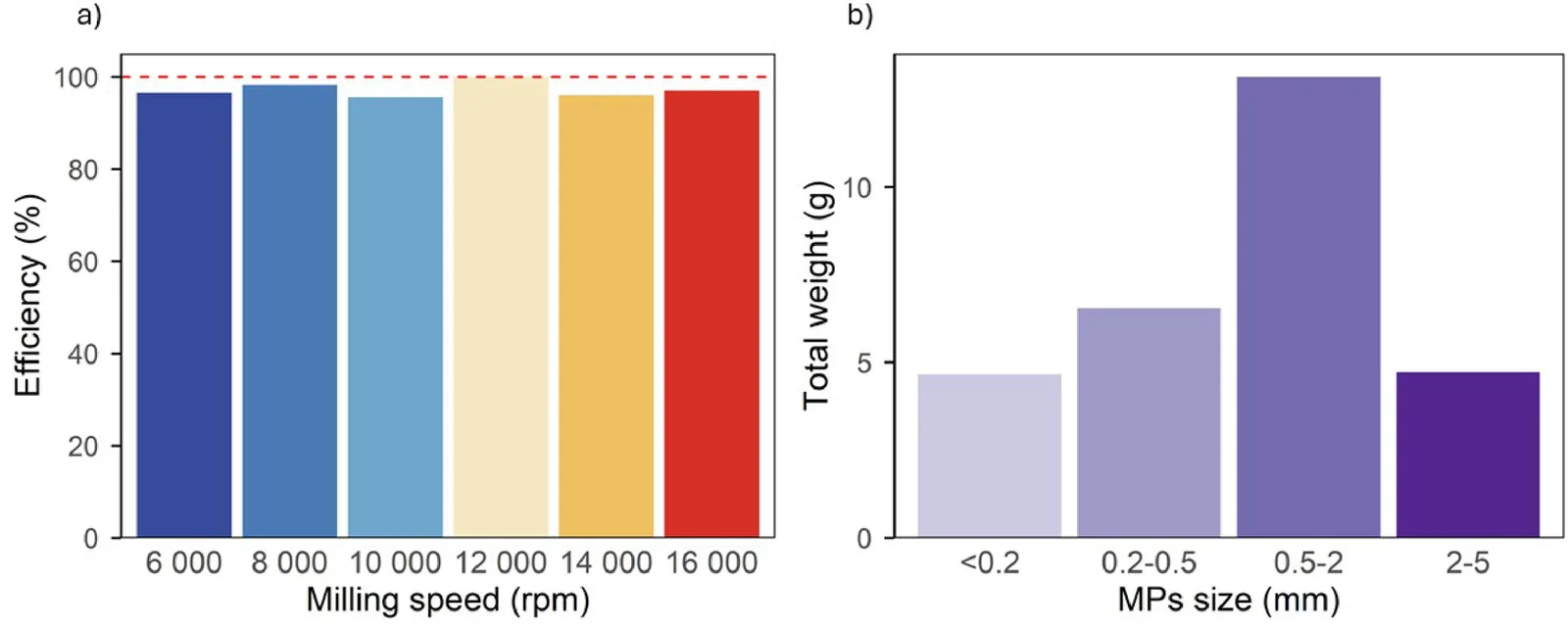
a) Percentage of MPs weight recovered relative to the initial BDM mass added, after milling across all tested speeds; b) Size distribution of the total MPs produced for all milling speeds combined.

**Fig. 3:**
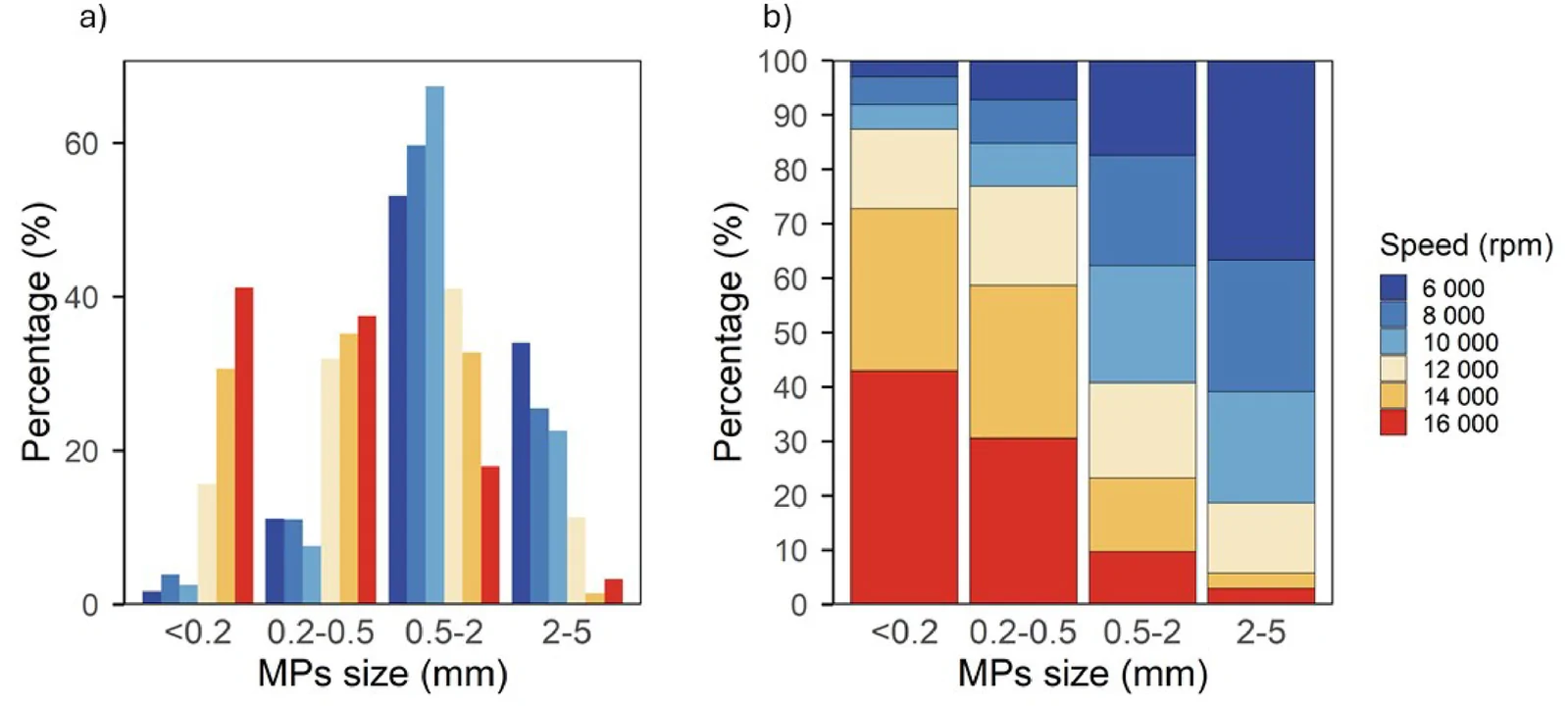
a) Size distribution of the MPs produced (%, w/w) according to the milling speed; b) Relative percentage (w/w) size distribution across milling speeds.

The following results were obtained with an initial weight of 5 g of BDM per milling speed. This method is an efficient and low-cost way to produce MPs of different sizes, up to 5 mm, from BDM, using a mechanical mill. Milling efficiency, defined as the percentage of MPs weight recovered after milling relative to the initial BDM mass, was consistently high across all tested speeds, ranging from 96.02% (14,000 rpm) to 100% (12,000 rpm) (Fig. 2a). A high proportion of MPs were obtained in the 0.5–2 mm fraction when considering all speeds together (Fig. 2b), and also for each individual speed (Fig. 3a).

**Fig. 4:**
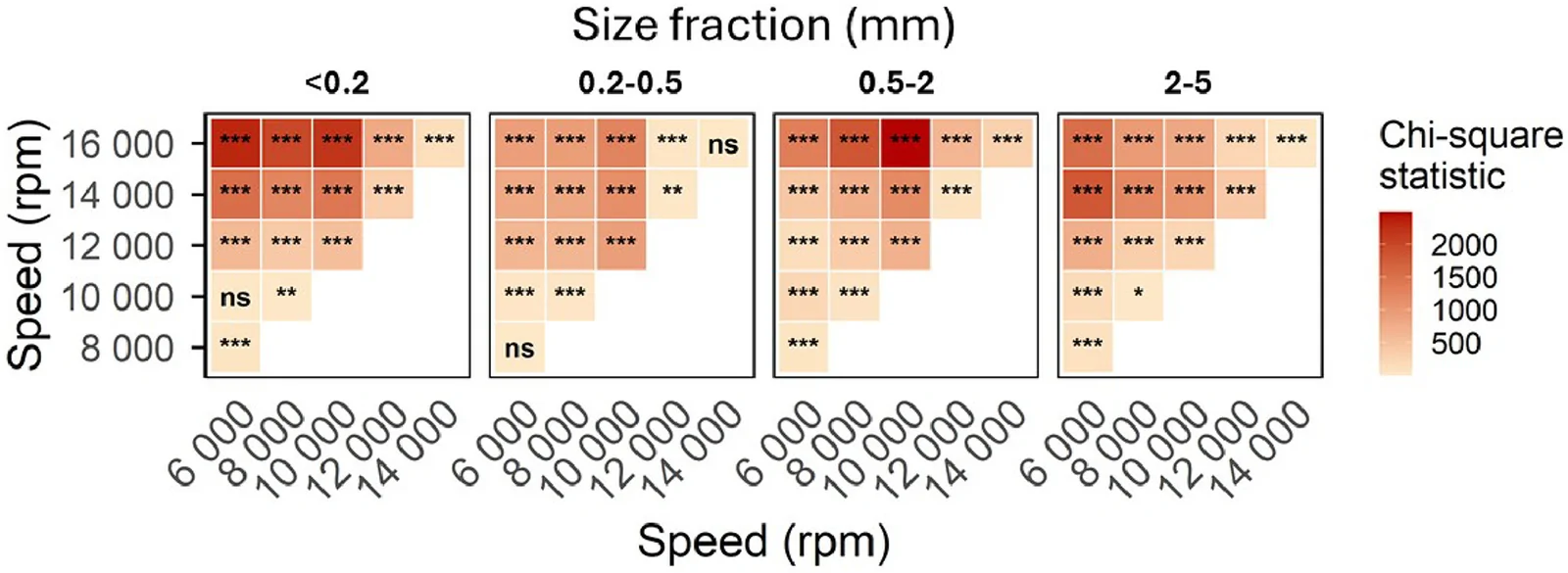
Pairwise chi-square statistics followed by Bonferoni post-hoc comparing MPs size distributions between milling speeds, for each size fraction. ns – non-significant; * - *p* < 0.05; ** - *p* < 0.01; *** - *p* < 0.001.

**Fig. 5:**
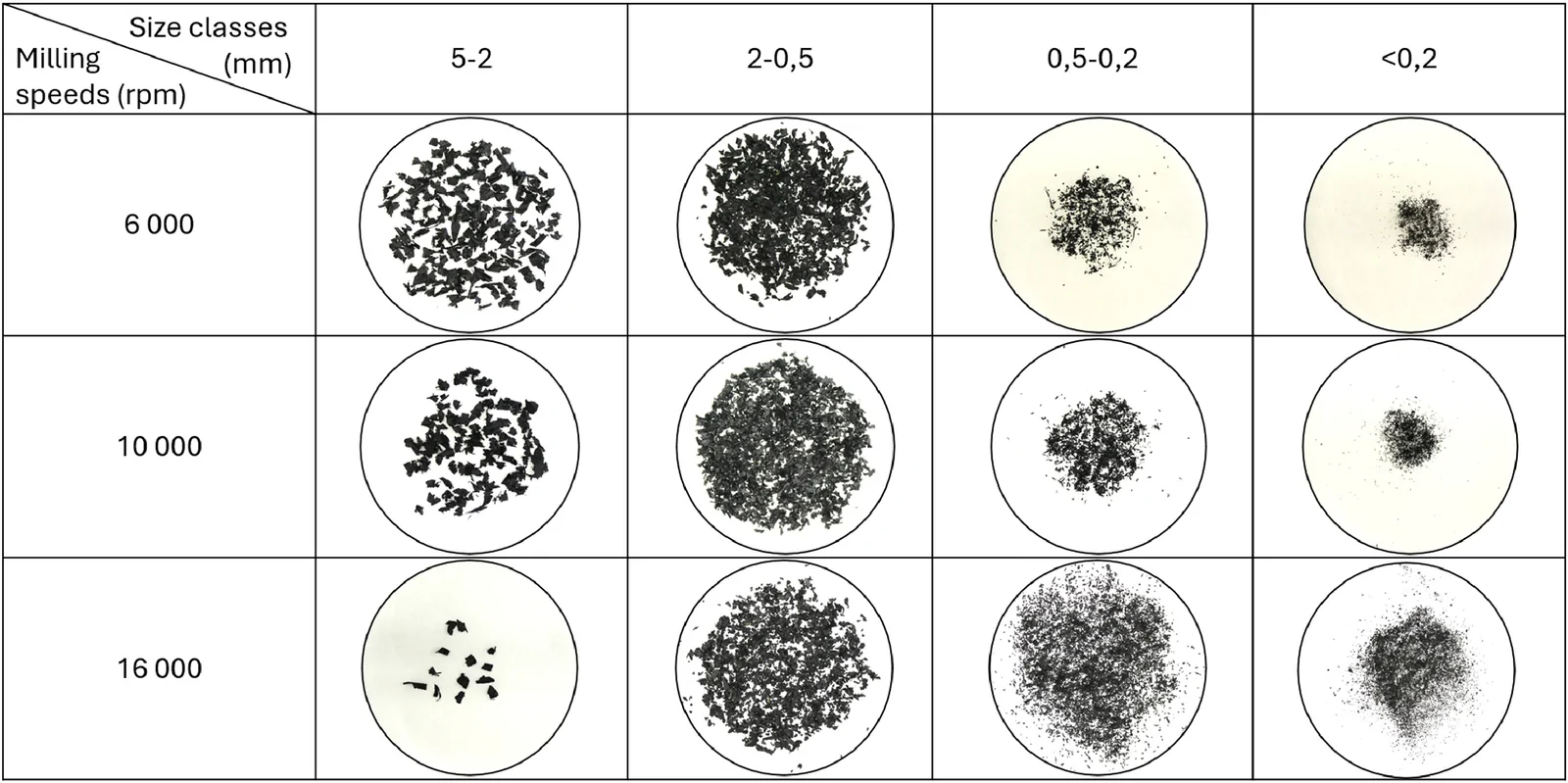
Visual representation of the BMD-derived MPs size distribution (% w/w) for the low, medium and high milling speeds (respectively 6000, 10 000 and 16,000 rpm).

The milling speed influences the MPs size distribution (Fig. 3a). The proportion of smaller MPs increases with higher milling speeds. The production of MPs < 0.2 mm increases by 25-fold when the speed is 16,000 rpm compared to 6000 rpm. Conversely, there were 10 times more MPs in 2–5 mm fraction at 6000 rpm compared to 16,000 rpm. At 10,000 rpm, the smallest particle measured had a size of 0.052 mm. The relative size distribution (Fig. 3b) highlights that higher milling speeds produce a greater proportion of smaller MPs. Pairwise chi-squared tests followed by Bonferoni post-hoc within each size fraction confirmed that the size distribution differed significantly between milling speeds (Fig. 4). The milling speed has a more pronounced effect on the smallest size fraction (<0.2 mm). Nevertheless, for the 0.2–0.5 mm fraction, adjacent milling speeds are not always significantly different (6000 vs. 8000 rpm and 14,000 vs. 16,000), indicating that speeds must be sufficiently distinct to produce meaningfully different size distributions. These differences in particle size distributions for the three optimal speeds can also be visually observed (Fig. 5). This method allows selection of the milling speed to produce the highest quantity of MPs of the specific sizes required by the intended experiments, e.g., ecotoxicity testing or soil mobility experiments.

Cryogenic milling has already shown its efficiency to mill BDM [15]. At a laboratory scale, cryomilling requires the use of specific vials with a capacity of up to 10 g of polymer (6875 Freezer/Mill High-Capacity Cryogenic Grinder) whereas the mechanical mill used here does not require vials and has a volume capacity of 300 mL that can host up to 30 g of the material. Moreover, the cryogenic mill requires 10 to 15 L of liquid nitrogen only to cool down the device, whereas with this mechanical milling only 300 mL of liquid nitrogen is required for milling 5 g of BDM pieces.

The methodology has also been followed with larger quantities of Mater-Bi® mulch. Within five hours, 600 g of MPs were obtained, with the same overall size distribution (see Fig. S1 in the supplementary material). Hence, large quantities of MPs can be produced efficiently, with size fractions driven by the milling speed, at low liquid nitrogen consumption.

The method was also successfully tested with other commercially available PBAT and PBAT/PLA BDM (Ecovio®, BASF, Germany; Bioflex®, FKUR, Germany), each producing very similar size distributions (see Fig. S2 in the supplementary material). PBAT- and PBAT/PLA-based BDMs are among the most widely used materials for agricultural mulching whereas BDMs based on other polymers, such as polybutylene succinate (PBS) and polybutylene succinate-co adipate (PBSA), are used considerably less frequently. Although these less commonly used BDMs were not investigated in the present study, the procedure is expected to be applicable to them provided that their relevant physical properties, particularly elongation at break, impact strength and glass-transition temperature, are sufficiently similar to those of the PBAT-based mulches tested here. Nevertheless, the variations in their properties may affect their milling behaviour and, particularly the MPs size distribution obtained at different milling speeds.

## Limitations

The timing during the milling is crucial to ensure that the BDM sheets remain frozen and that the mill itself remains cold during the milling; the steps are to be followed as described to keep the BDM frozen.

This method was developed with a range of commercially available BDMs. It may not work with low-density PE mulches.

## CRediT authorship contribution statement

**Mathilde Henrion:** Conceptualization, Formal analysis, Investigation, Data curation, Writing – original draft, Writing – review & editing. **Ana M. Pelacho:** Conceptualization, Methodology, Investigation, Data curation, Writing – review & editing, Supervision, Project administration. **Iseult Lynch:** Conceptualization, Writing – review & editing, Supervision. **Lluis Martin-Closas:** Methodology, Investigation, Resources, Writing – review & editing, Supervision, Project administration, Funding acquisition.

## Declaration of competing interest

The authors declare that they have no known competing financial interests or personal relationships that could have appeared to influence the work reported in this paper.

## Data Availability

Data will be made available on request.

## Appendix Supplementary materials

Image, application 1
