# Supplementary material for: High-yield large scale production of size-customized microplastics from biodegradable plastic mulches via variable-speed mechanical milling
Source: MethodsX. 2026 Aug 21;17:104110. doi: 10.1016/j.mex.2026.104110 (PMC13570288; doi:10.1016/j.mex.2026.104110)
Supplement: Supplementary file 1 [file mmc1.docx]

Supplementary material – MethodsX

**High-yield large scale production of size-customized microplastics from biodegradable plastic mulches via variable-speed mechanical milling**

Mathilde Henrion^a^, Ana M. Pelacho^a^, Iseult Lynch^b^, Lluis Martin-Closas^a,*^

^a^ Dpt. Agricultural and Forest Science and Engineering, University of Lleida, Av Alc. Rovira Roure, 191, *25*198 Lleida, Spain

^b^ School of Geography, Earth and Environmental Sciences, University of Birmingham, B15 2TT Birmingham, UK

Fig. S1. Production of a large amount of MPs (600g in total). a) Size distribution of the total MPs produced for all milling speeds combined; b) Relative percentage (w/w) size distribution across milling speeds; c) Size distribution of the MPs produced (%, w/w) according to the milling speed from two batches, followed by Spearman correlation statistics per size fraction.

Fig. S2. Particle size distribution of three commercially available biodegradable mulches (15 µm thick) milled at 10 000 rpm following the same procedure as described in the main text. Ecovio® is composed of PBAT and PLA plus additives. Bioflex® is made from PBAT and PLA blends coming from corn or sugar cane plus additives.
